# Prenatal diagnosis of a fetal harlequin ichthyosis

**DOI:** 10.1007/s00404-023-07164-9

**Published:** 2023-07-25

**Authors:** Tobias Spingler, Cornelia Wiechers, Markus Hoopmann, Karl Oliver Kagan

**Affiliations:** 1 Department of Obstetrics and Gynaecology, University of Tuebingen, Calwerstrasse 7, 72076 Tübingen, Germany; 2 Department of Neonatology, University of Tuebingen, Tübingen, Germany

**Fig. 1:**
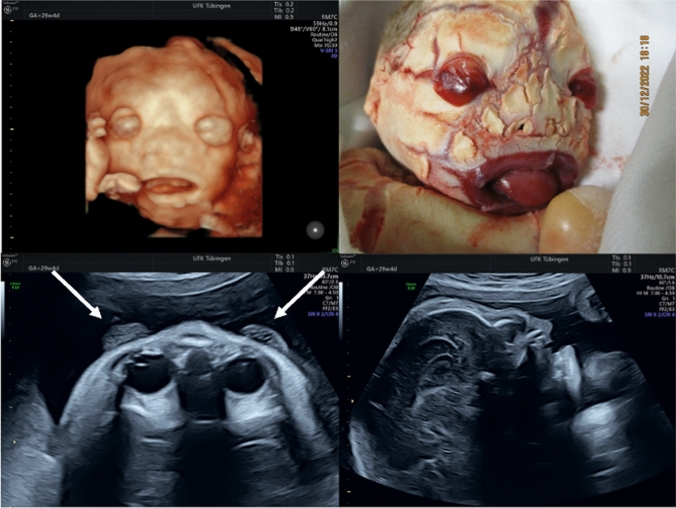
3D image of the face with an open mouth (upper left), profile (lower right), and ectropion (denoted by arrows) (lower left). The upper right is a corresponding postnatal image

A 24-year-old woman (gravida 2, para 1) was referred at 30 week gestation due to an abnormal fetal profile on prenatal ultrasound and reduced fetal movements. Our ultrasound examination showed the typical dysmorphic facial features of harlequin ichthyosis (HI) including ectropion of the eyelids, outward turning of the lip (eclabium) with an open mouth, and the typical profile. In addition, the joints were immobile and the position of the fingers and toes was abnormal. Interestingly, respiratory movements were still visible. After counseling, the couple decided to terminate the pregnancy Fig. 1.

The figure includes some typical features of HI on ultrasound: 3D image of the face with an open mouth (upper left), profile (lower right), and ectropion (denoted by arrows) (lower left). The upper right is a corresponding postnatal image.

HI is a severe and rare autosomal recessive ichthyosis with a prevalence of approximately 1 in 300 000 births [1]. The disease is caused by a mutation in the lipid transporter adenosine triphosphate-binding cassette A 12 (ABCA12) [2]. Prenatally, typical signs of the disease, such as ectropion, eclabium, dense suspended particles in the amniotic fluid, rudimentary ears, and contractures, are not detectable until the second trimester, so early detection in pregnancy is not possible. Affected children also show armor-like thickening of the skin, which can lead to pseudocontractures and necrosis.

Therapy for HI includes intensive neonatal care and administration of retinoids such as oral acitretin within the first 7 days of life. Even though this therapeutical approach improves the survival rate, 75% of the neonates still die within the first 3 months of life, usually due to sepsis or respiratory failure [3, 4].
